# The Effect of Molecular Properties on Active Ingredient Release from Electrospun Eudragit Fibers

**DOI:** 10.3390/pharmaceutics10030103

**Published:** 2018-07-24

**Authors:** Kieran Burgess, Heyu Li, Yasmin Abo-zeid, Fatimah, Gareth R. Williams

**Affiliations:** UCL School of Pharmacy, University College London, 29-39 Brunswick Square, London WC1N 1AX, UK; kieran.burgess.16@ucl.ac.uk (K.B.); heyu.li@ucl.ac.uk (H.L.); y.abozeid@ucl.ac.uk (Y.A.-z.); fatimah.14@ucl.ac.uk (F.)

**Keywords:** electrospinning, Eudragit, nanofibers, drug release

## Abstract

The formation of nanoscale fibers from pH-sensitive polymers is a route which has been widely explored for targeted drug delivery. In particular, the Eudragit L100 and S100 families of polymers have received significant attention for this purpose. However, while in some cases it is shown that making drug-loaded Eudragit polymers effectively prevents drug release in low-pH media where the polymer is insoluble, this is not always the case, and other studies have reported significant amounts of drug release at acidic pHs. In this study, we sought to gain insight into the factors influencing the release of active ingredients from Eudragit S100 (ES100) fibers. A family of materials was prepared loaded with the model active ingredients (AIs) benzoic acid, 1-naphthoic acid, 1-naphthylamine, and 9-anthracene carboxylic acid. Analogous systems were prepared with an AI-loaded core and an ES100 sheath. The resultant fibers were smooth and cylindrical in the majority of cases, and X-ray diffraction and differential scanning calorimetry showed them to comprise amorphous solid dispersions. When AI release from the monolithic fibers was probed, it was found that there was significant release at pH 1 in all cases except with 9-anthracene carboxylic acid. Analysis of the results indicated that both the molecular weight of the AI and its acidity/basicity are important in controlling release, with lower molecular weight AIs and basic species released more quickly. The same release trends are seen with the core/shell fibers, but AI release at pH 1 is attenuated. The most significant change between the monolithic and core/shell systems was observed in the case of 1-naphthylamine. Mathematical equations were devised to connect molecular properties and AI release under acidic conditions.

## 1. Introduction

Electrospinning is a technique which has attracted great attention in the pharmaceutical technology field [1,2]. It most commonly involves the preparation of a polymer solution in a volatile solvent. This is loaded into a syringe and is slowly ejected through a narrow bore needle (the spinneret). A high voltage power supply is used to charge the needle, and the solution expelled towards a grounded collector plate. The application of the electrical energy causes drawing of the polymer solution into a fine jet, and ultimately results in the production of fibers with diameters typically on the nanoscale. The inclusion of a drug molecule in the solution generally yields drug-loaded fibers in the form of amorphous solid dispersions. In the simplest embodiment of the experiment, a single liquid is processed, but more advanced derivatives including coaxial electrospinning (which uses two needles nested concentrically one inside the other to process two liquids) and triaxial spinning (three needles, three solutions) have also been reported. The use of coaxial spinnerets results in core/shell structures, and triaxial spinnerets give three-compartment architectures.

Electrospun nanofibers have been explored for a wide range of drug delivery applications, including preparing fast-dissolving oral drug delivery systems designed for very rapid release in the mouth [3,4], extended release systems allowing the drug cargo to be freed over a number of hours or weeks [5,6,7,8], and systems able to respond to external stimuli such as temperature [9,10,11,12]. Given that the pH of the human gastrointestinal tract varies from 1–3 in the stomach, 6–8 in the small intestine, and 4–7 in the large intestine, materials able to respond to changes in pH are particularly useful for oral delivery systems. A range of polymers exist which are selectively soluble above or below a particular pH. One clinically used family of such polymers is the Eudragits, methacrylate-based polymers with tunable pH sensitivity. Eudragit L100-55 is soluble above pH 5.5, L100 above pH 6, and S100 above pH 7. This allows different sections of the intestinal tract to be targeted depending on the polymer chosen to fabricate a formulation. A number of authors have explored the electrospinning of Eudragits, as well as other pH-sensitive polymers such as shellac [13,14].

The studies in the literature exploring Eudragit have investigated both monolithic fibers from monoaxial electrospinning and core/shell materials. Shen et al. were the first to electrospin Eudragit, preparing L100-55 fibers loaded with diclofenac sodium [15]. They found that drug release at pH 1.0 was below 3%, confirming that pH-sensitive fibers can be produced. Other studies have built on this and reported similar conclusions, for instance with Eudragit fibers containing mebeverine HCl [16], ketoprofen [17], indomethacin [18] and helicid [19]. However, it is not always the case that simply making a drug-loaded Eudragit fiber formulation prevents drug release in the acidic pHs typical of the stomach. For instance, Karthikeyan et al. reported blend fibers of zein and Eudragit S100 (ES100) loaded with pantoprazole and aceclofenac gave 25% release of the latter after 2 h immersion in 0.1 M HCl [20]. The same has been shown to be true for spironolactone-loaded Eudragit FS fibers (up to 30% release at pH 1.2) [21], and 5-fluorouracil (5-FU)-loaded ES100 fibers, which showed some 80% release at pH 1 [22].

Analogous findings have been found when preparing core/shell systems. Whilst in some instances it is reported that drug release at pH 1–3 can be virtually completely obviated in fibers with a Eudragit shell and drug-loaded core (for instance containing a Gd-based contrast agent or indomethacin [23,24]), similar fibers containing 5-FU in the core release up to 70% of their drug loading under these conditions [22]. The reasons behind the different findings reported in the literature are not completely clear, but it seems that both the molecular weight and acidity or basicity of the drug incorporated are important. Most recently, Jia et al. prepared fibers with an ES100 shell and a poly(ethylene oxide) core containing either mebeverine HCl or indomethacin (model basic and acidic drugs with similar molecular weights (MWs)) [25]. It was observed that release of both the basic mebeverine and acidic indomethacin at pH 1.2 was restrained by the presence of the ES100 shell, but that there was a noticeably greater extent of release in the former case (up to 20%) than the latter (ca. 1%).

**Figure 1:**
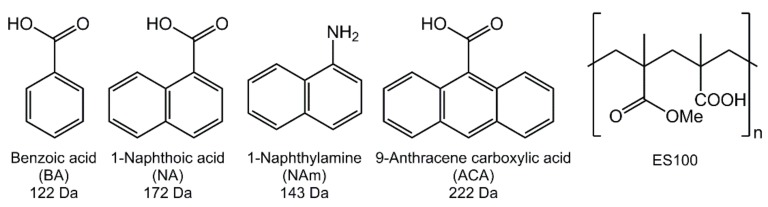
The chemical structures of the active ingredients used in this work and their molecular weights, together with the structure of the polymer ES100. BA: Benzoic acid; NA: 1-naphthoic acid; NAm: 1-Naphthylamine; ACA: 9-anthracene carboxylic acid; ES100: Eudragit S100.

In this work, we sought to obtain a fundamental understanding of the factors underlying drug release from Eudragit fibers at acidic pHs. To do this, we assembled a training set of four model active ingredients (AIs; Figure 1). While none has any direct applications in drug delivery, the incremental variation in their structures is ideal for a fundamental study of this type. Benzoic acid (BA), 1-naphthoic acid (NA) and 9-anthracene carboxylic acid (ACA) all contain one carboxylic acid functional group and have similar pK_a_s (BA: 4.20; NA: 3.67; ACA: 3.68), but the number of aromatic rings in the series increases from one to three. 1-Naphthylamine (NAm) has an identical structure to NA except that the carboxylic acid is replaced by an amine group; the pK_a_ of NAm conjugate acid is very similar to that of NA at 3.92, and thus the influence of acidity/basicity can be elucidated through this molecule pair.

A series of ES100-based fibers was prepared containing the training set AIs, using both monoaxial electrospinning to generate monolithic composites and also coaxial spinning to produce core/shell systems with the AI confined to the core. The fibers were subject to a detailed examination of their physicochemical properties, and AI release explored at pH 1.0 and 6.8 following pharmacopoeia protocols. Correlations between molecular properties and the release profiles were sought.

## 2. Materials and Methods

### 2.1. Materials

Benzoic acid (BA), 1-naphthoic acid (NA), 1-naphthylamine (NAm), 9-anthracene carboxylic acid (ACA), absolute ethanol and dimethylacetamide (DMAc) were purchased from Sigma-Aldrich (Gillingham, UK). Eudragit S100 (ES100) was a kind gift from Evonik GmbH (Darmstadt, Germany). Analytical grade hydrochloric acid and trisodium phosphate dodecahydrate were obtained from Fisher Scientific (Loughborough, UK). All water was deionised before use.

### 2.2. Methods

#### 2.2.1. Monoaxial Electrospinning

Following a series of optimisation experiments, a solvent mixture of ethanol/water/DMAc (15/1/4 *v*/*v*/*v*) was selected as the most appropriate for electrospinning. A series of solutions was then prepared for monoaxial electrospinning (see Table 1). 1.2 g of ES100 and 0.1 g of the AI of interest were dissolved in 10 mL of the ethanol/water/DMAc solvent system. These were magnetically stirred for a minimum of 24 h to ensure a homogenous solution was formed.

The solutions were loaded into a 5 mL Terumo syringe, with great care taken to avoid the formation of bubbles. The syringe was then fitted with a blunt-tipped metal needle (internal diameter 0.61 mm; Nordson EFD, Aylesbury, UK), and the positive electrode of a high-voltage power supply (HCP 35–35000, FuG Elektronik, Schechen, Germany) connected to the tip of the needle via a crocodile clip. The grounded collector comprised a flat piece of steel coated with aluminium foil. Liquid was dispensed using a syringe pump (KDS100, Cole Parmer, London, UK). Electrospinning was performed at ambient conditions (25 ± 3 °C; relative humidity 38 ± 6%), and processing parameters were as follows: voltage 16 kV; flow rate 0.5 mL h^−1^; collection distance 18 cm. After fabrication, the fiber products were stored in a desiccator over silica beads.

#### 2.2.2. Coaxial Electrospinning

A 12% *w*/*v* Eudragit S100 solution in ethanol/water/DMAc (15/1/4 *v*/*v*/*v*) was used as the shell liquid for coaxial electrospinning. The core solutions were the same as those used for monoaxial electrospinning (see Table 1). The applied voltage for coaxial spinning was 21 kV, the collection distance 18 cm, and experiments were performed under ambient conditions (25 ± 3 °C; relative humidity 38 ± 6%). The core and sheath solutions were independently dispensed through a coaxial spinneret (Linari Engineering, Pisa, Italy) using two separate KDS100 syringe pumps. The spinneret had internal/external diameters for the inner needle of 0.51/0.83 mm and for the outer needle at 1.37/1.83 mm. The core and shell flow rates were 0.4 and 0.8 mL/h respectively. After production, the fiber products were stored in a desiccator over silica beads.

### 2.3. Characterisation

#### 2.3.1. Electron Microscopy

Small samples (ca. 0.5 × 0.5 cm) were cut from each fiber mat for scanning electron microscopy (SEM). These were sputter coated with gold and then imaged using a Quanta 200F instrument (FEI, Hillsboro, OR, USA). The fiber diameters were quantified at 100 points for each sample, using the ImageJ software (v1.48; National Institutes of Health, Bethesda, MD, USA). The coaxial materials were also studied with transmission electron microscopy (TEM) on a CM 120 Bio-Twin instrument (Philips, Amsterdam, The Netherlands). For this, fibers were directly spun onto carbon-coated TEM grids (TAAB, Aldermaston, UK).

#### 2.3.2. Physical Form Characterisation

X-ray diffraction (XRD) patterns were collected on a MiniFlex 600 diffractometer (Rigaku, Tokyo, Japan) supplied with Cu Kα radiation. Data were collected over the 2θ range 3 to 35° at a rate of 5° min^−1^. Differential scanning calorimetry (DSC) was performed on a Q2000 instrument (TA Instruments, New Castle, DE, USA). Samples weighing between 4–7 mg were placed into T-zero aluminium pans, sealed, and pinholed. Samples were equilibrated at 0 °C, heated to 100 °C at 10 °C min^−1^, and then cooled to 0 °C again. A second heating run (to 200 °C in most cases) was finally performed at 10 °C min^−1^. All DSC experiments were undertaken under a nitrogen purge of 50 mL min^−1^. IR spectra were obtained with a Spectrum 100 spectrometer (Perkin Elmer, Waltham, MA, USA) over the wavenumber range 650–4000 cm^−1^ and with resolution of 1 cm^−1^.

#### 2.3.3. Active Ingredient Loading

After the fibers had been left to dry to remove any residual solvent, samples (ca. 10 mg) were cut from each sample and dissolved in a mixture of ethanol/water/DMAc (15/1/4 *v*/*v*/*v*). Calibration curves for each active ingredient (AI) were prepared using a 7315 spectrophotometer (Jenway, Stone, UK), and the AI loading determined (*n* = 3).

### 2.4. Dissolution Studies

Calibration curves were constructed for each AI at pH 1.0 (0.1 M HCl) and 6.8 (phosphate buffered saline; PBS) with the aid of a 7315 spectrophotometer (Jenway, Stone, UK). Dissolution studies were then undertaken following the USPII method on an automated instrument (Caleva, Dorchester, UK). The dissolution vessels were initially charged with 750 mL of 0.1 M HCl and equilibrated at 37 ± 0.5 °C under stirring at 50 rpm. Lids on each vessel prevented evaporation. Capsule sinkers were manually filled with ca. 60 mg of a fiber mat and placed into the vessel. Aliquots (5 mL) were periodically withdrawn from the vessels, and replaced with 5 mL of preheated 0.1 M HCl to maintain a constant volume. After 2 h of operation, 250 mL of preheated 0.2 M trisodium phosphate dodecahydrate was added to each vessel, yielding 1 L of a buffer at pH 6.8. Again, 5 mL aliquots were withdrawn at specific time points, and replenished with 5 mL of preheated PBS at pH 6.8. The AI concentrations in each aliquot were quantified with a 7315 spectrophotometer (Jenway, Stone, UK), and cumulative release percentages calculated from these.

## 3. Results

### 3.1. Monolithic Fibers

**Figure 2:**
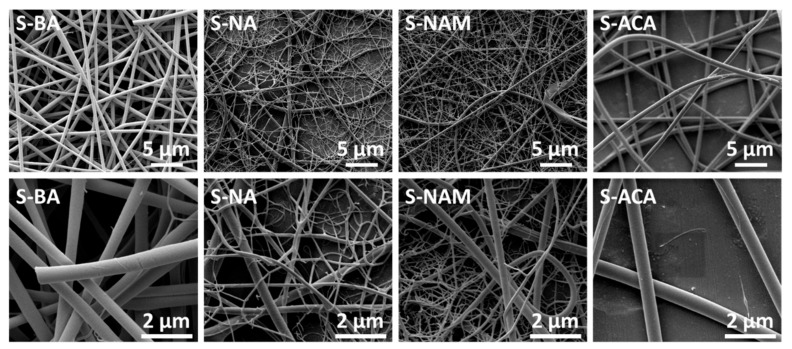
SEM images of the monolithic fibers (top: 10,000× magnification; bottom 40,000× magnification).

Scanning electron microscopy (SEM) images of the monolithic fibers are presented in Figure 2. In all cases, cylindrical fibers have clearly been formed. In the cases of S-BA and S-ACA, the fibers are homogenous and well formed, with diameters around 500–600 nm (see Table 1). In contrast, the S-NA and S-NAM materials clearly contain two populations of fibers, with a number of very small branched fibers visible, likely due to there being some jet instability during spinning. As a result, the average diameter is rather smaller for these fibers, at 129 ± 102 nm for S-NA and 214 ± 105 nm for S-NAM, and there is a proportionally greater deviation in the diameters.

**Figure 3:**
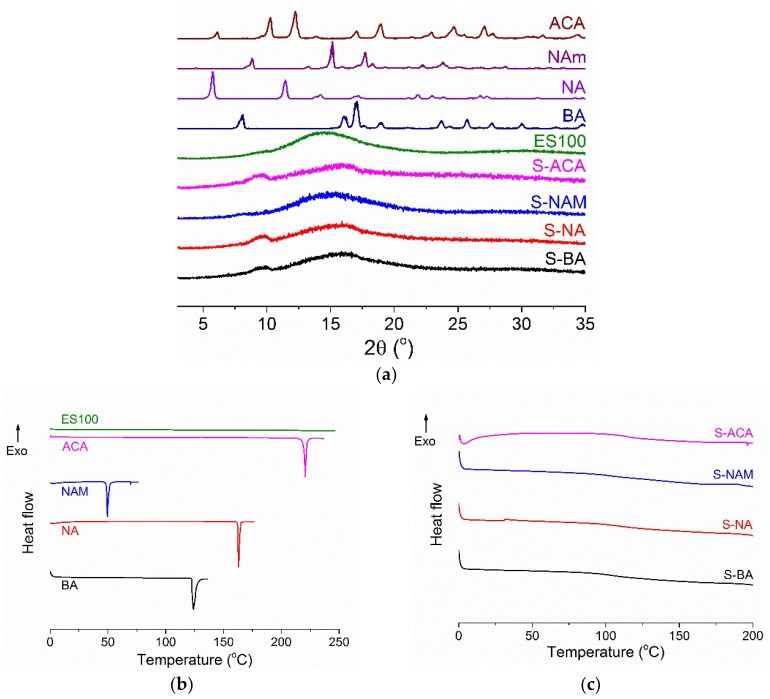
(**a**) XRD data and DSC data on (**b**) the raw materials and (**c**) formulations from monoaxial electrospinning. The data in (**a**) are normalized for ease of comparison.

The physical form of the AI in the fibers was explored using XRD and DSC (Figure 3). The XRD data in Figure 3a demonstrate that the AIs are crystalline materials, with numerous Bragg reflections visible in their patterns. ES100 is amorphous, and only a broad halo is present in its pattern. No Bragg reflections can be observed for the fibers, showing them to be amorphous solid dispersions. The DSC data (Figure 3b,c) concur with these findings. While the AIs exhibit sharp melting endotherms in their thermograms, these are lacking for ES100 and all the fiber formulations. It should be noted that the DSC experiments were stopped at 200 °C with the fibers, since in preliminary experiments degradation was observed to start just above this temperature (data not shown). Thus, the melting of ACA would not be seen in the data even if crystalline material were present. However, T_g_ events are present between 100 and 125 °C for all the fibers, confirming their amorphous nature.

**Figure 4:**
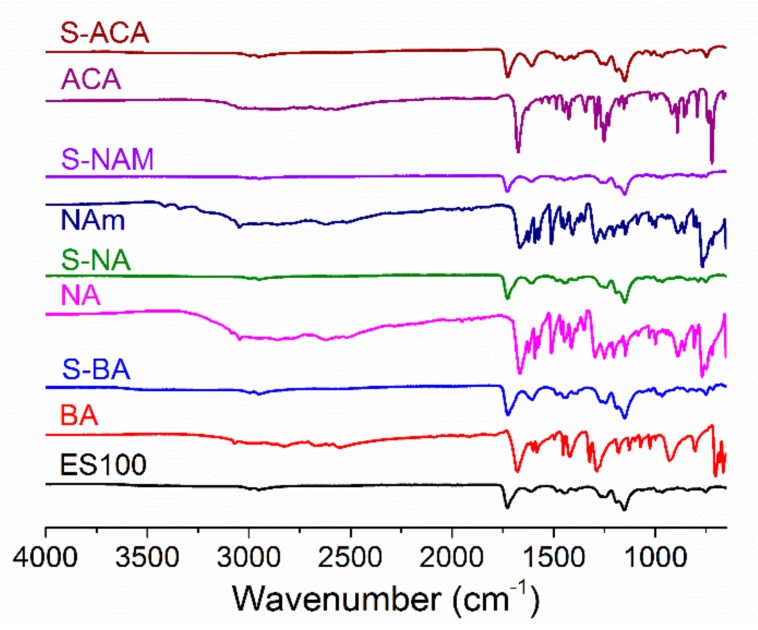
IR spectra of the monolithic fibers.

IR spectra are given in Figure 4. ES100 has characteristic peaks at 2950–3000 cm^–1^ (CH_2_ stretching), 1727 cm^–1^ (C=O stretching), and 1150–1275 cm^−1^ (C–O–C stretching). The spectra of the AI-loaded fibers are very similar to that of ES100, as expected given that the AI loading is relatively low (theoretical loading: 7.69%). However, some subtle changes can be observed. The C=O stretch of pure BA is found at 1678 cm^−1^, and merges with the ES100 C=O stretch in the S-BA fibers to form a single peak at 1725 cm^−1^. This shift might be attributed to the formation of intermolecular bonding interactions (e.g., H-bonding) between BA and ES100. Similarly, NA and ACA display a C=O stretch at 1667 and 1674 cm^−1^ while the S-NA and S-ACA fibers display single C=O bands at 1727 and 1726 cm^−1^ respectively. No shoulders are visible on these peaks, suggesting again that intermolecular interactions cause the bands to merge. In the case of S-NAM, the N–H stretching vibrations of NAm (at 3411 and 3341 cm^−1^) are not visible in the fibers, which might be indicative of intermolecular bonding or simply the low AI loading in the formulations.

The AI loading (Table 1) is close to 100% of the theoretical content (>85% in all cases). The differences observed can be attributed to losses of AI during electrospinning (e.g., through small amounts of precipitation or adherence to the syringe walls), the presence of some residual solvent in the fibers, or small inaccuracies in the quantification method.

### 3.2. Core/Shell Fibers

**Figure 5:**
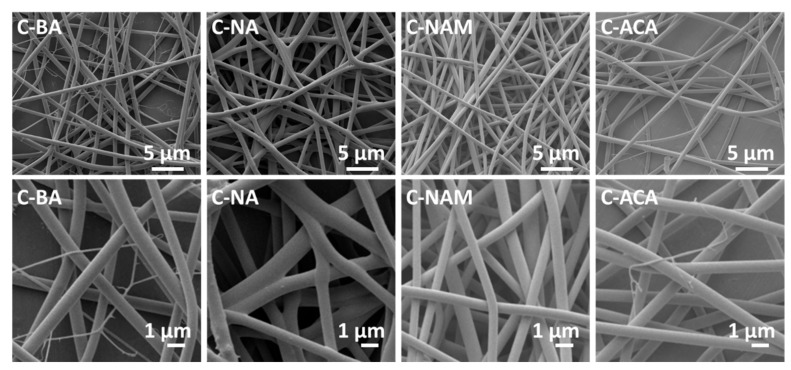
SEM images of the coaxial fibers.

Core/shell fibers were prepared with an ES100 shell and a core comprising ES100 and the AI. SEM images of these are given in Figure 5. The fibers are, in general, smooth and homogeneous, with average diameters of around 550–700 nm as detailed in Table 1. For C-BA and C-ACA there are a few very fine fibers present as well as the bulk at ca. 600 nm. For C-NA, what appear to be particles can be seen on the fiber surfaces, suggesting some phase separation may have occurred.

**Figure 6:**
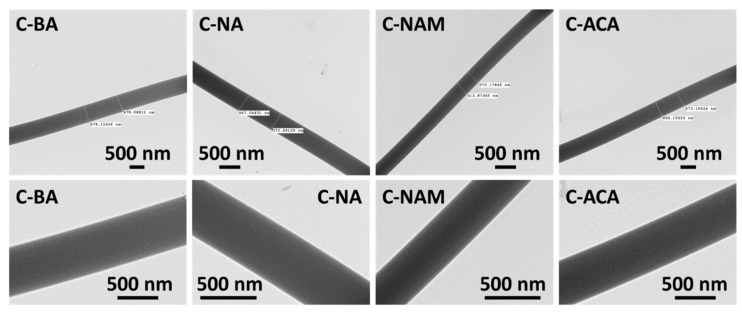
TEM images of the coaxial fibers.

TEM images of the fibers from coaxial electrospinning are depicted in Figure 6. The two solutions used for the core and shell are very similar in their composition, and hence the contrast between the core and shell is not particularly distinct. However, on close inspection it is clear that the fibers have separate core and shell compartments.

**Figure 7:**
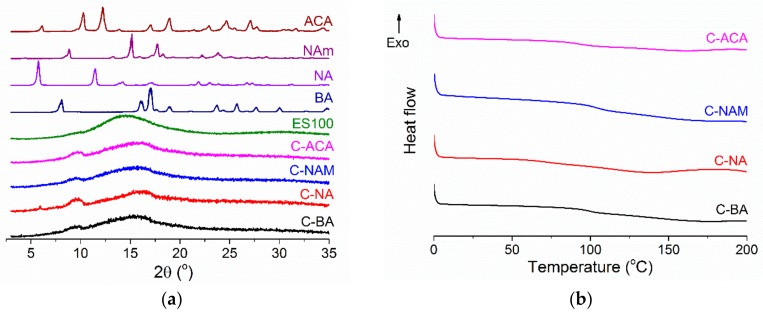
(**a**) XRD and (**b**) DSC data for the coaxial fibers. The data in (**a**) are normalized for ease of comparison.

The physical form of the AI in the fibers was probed using XRD and DSC (Figure 7). Similarly to the monolithic fibers, there is no evidence for any crystalline material being present in the products of coaxial electrospinning: the sharp Bragg reflections of the pure AIs are replaced by broad haloes in the XRD patterns of the fibers, consistent with amorphous AI-in-polymer dispersions. The DSC data are also typical of amorphous systems, with no melting endotherms visible but instead clear baseline changes corresponding to T_g_s.

**Figure 8:**
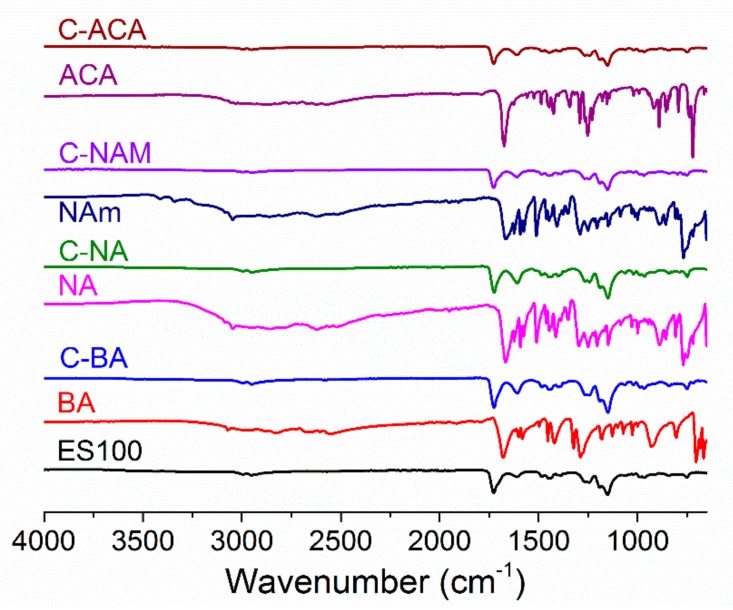
IR spectra of the coaxial fibers.

IR spectra of the core/shell fibers (Figure 8) again are very similar to that of pure ES100, except with some small shifts in peak position (for instance, the C=O peak shifts from 1727 cm^−1^ in ES100 to 1725 cm^−1^ in C-BA and C-NA). Given the low AI loadings of these fibers (theoretical loading: 2.70%) it is not possible to draw any firm conclusions relating to physical form from the IR spectra alone, but it appears that intermolecular bonding may be operational here too.

The AI loadings observed (see Table 1) are again close to 100% of the theoretical content (>80%), with the values close to those found in the monoaxial fibers.

### 3.3. AI Release

**Figure 9:**
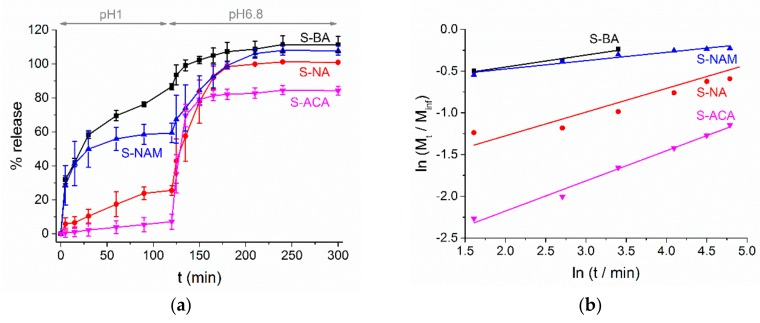
(**a**) AI release from the monolithic formulations, with data given as mean ± S.D. from three independent experiments; (**b**) fitting the Korsmeyer-Peppas model to the experimental data obtained at pH 1. Percentages are given relative to the theoretical AI loading in the fibers.

The AI release profiles for the monolithic fibers are shown in Figure 9a. It is clear that the simple fact of making fibers from ES100 does not prevent AI release at pH 1. This is because the AI is able to diffuse through the polymer matrix to reach solution. The extent to which this occurs depends on the nature of the molecule used as the AI: the amount of release in acidic pH decreases in the order S-BA (86.6 ± 1.9%) > S-NAM (59.4 ± 5.7%) > S-NA (25.5 ± 2.8%) > S-ACA (7.1 ± 4.5%). Both molecular weight (MW) and the acidity/basicity of the AIs are thus important: the lower MW BA (122 Da) releases more quickly and to a greater extent than the intermediate NAm (143 Da) and NA (172 Da) and higher MW ACA (222 Da). S-NAM releases markedly more than S-NA, presumably due to the basic nature of NAm favoring dissolution in the low pH environment in the former case.

This hypothesis is supported by a Korsmeyer-Peppas analysis of the release data at pH 1 (Figure 9b and Table 2). This model takes the form M_t_/M_inf_ = kt^n^, where M_t_ is the amount of AI released at time t, M_inf_ is the theoretical AI loading of the fibers, k is a rate constant, and n is an exponent providing information on the reaction mechanism. For all the monolithic fibers, n is <0.45, indicating that Fickian diffusion is the predominant reaction mechanism. The calculated k values fall in the same order as the extent of release data, as would be expected.

Considering the release observed at pH 6.8, all the formulations rapidly release the majority of their remaining AI content after the pH is elevated, with maximum release attained after ca. 60 min immersion in PBS. S-BA, S-NA and S-NAM all reach approximately 100% release, while for S-ACA the plot levels off at around 84%. This can be attributed to the larger MW and lower solubility of ACA compared to the other AIs, as well as the reduced entrapment efficiency noted for S-ACA (calculated to be 85% of the theoretical loading). There are insufficient datapoints for a Peppas analysis of the release data at pH 6.8 but the slopes of plots of ln (M_t_/M_inf_) vs. ln t are much steeper than at acidic pH, indicating that polymer swelling and dissolution are the dominant release mechanisms in neutral conditions.

**Figure 10:**
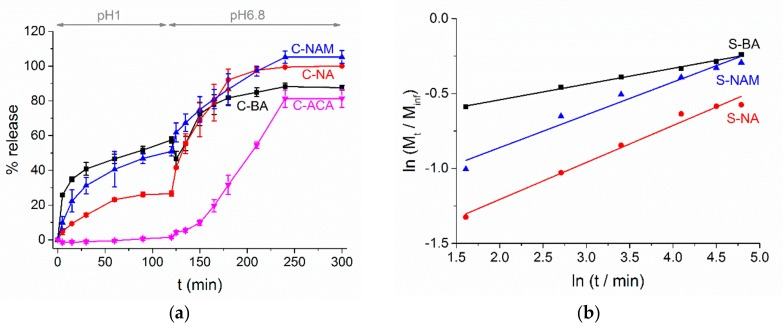
(**a**) AI release from the core/shell formulations, with data given as mean ± S.D. from three independent experiments; (**b**) fitting the Korsmeyer-Peppas model to the experimental data obtained at pH 1. Percentages are given relative to the theoretical AI loading in the fibers.

AI release from the coaxial fibers shows similar trends to the monoaxial analogues (Figure 10a). The presence of a blank ES100 shell clearly reduces the amount of AI release seen at pH 1, with C-BA releasing only 57.4 ± 1.7% of its AI loading, cf. 86.6 ± 1.9% in the case of the monolithic systems. Similar results are seen for C-NAM vs. S-NAM (50.9 ± 2.8% and 59.4 ± 5.7% release, respectively) and the ACA-loaded formulations (C-ACA 1.5 ± 0.8%; S-ACA 7.1 ± 4.5%). C-NA and S-NA behave very similarly (C-NA 26.6 ± 1.5%; S-NA 25.5 ± 2.8%). It is evident in all cases bar ACA that a significant proportion of the AI is freed into acidic solution, even though the ES100 sheath is insoluble.

Korsmeyer-Peppas analysis of the pH 1 release data (Figure 10b and Table 2) indicates that, as for the monolithic materials, AI release is diffusion controlled, with n < 0.45. Looking at the rate constants (k), only for C-NAM is there any noticeable change from the monolithic fibers. This is potentially attributable to the basic nature of NAm: NAm has a pKa of 3.92, and so will be fully ionized at pH 1. This will cause it to be highly soluble, providing a thermodynamic driver for the molecules to diffuse out of the polymer matrix and into solution. The other three AIs are acidic, and will be 100% unionized at pH 1. It should be noted that the Korsmeyer-Peppas equation is really only suitable for release from monolithic systems however, and thus some caution must be exercised in considering the results.

When the pH is raised to 6.8, as for the monolithic materials the rate of AI release accelerates noticeably. Both C-NAM and C-NA release 100% of their AI cargo, while C-ACA releases around 81% of the theoretical loading (again consistent with the lower entrapment efficiency of this formulation (91%)). Interesting, C-BA behaves more similarly to the C-ACA material than the naphthalene analogues. It is not obvious why this should be, but it may be that BA forms strong H-bonds with the ES100 carrier as it dissolves, resulting in the formation of AI-polymer aggregates which are removed by filtration before the release percentage is quantified. The maximum extent of release in all cases is reached more slowly than for the monolithic systems, at around 2 h after the elevation of pH.

**Figure 11:**
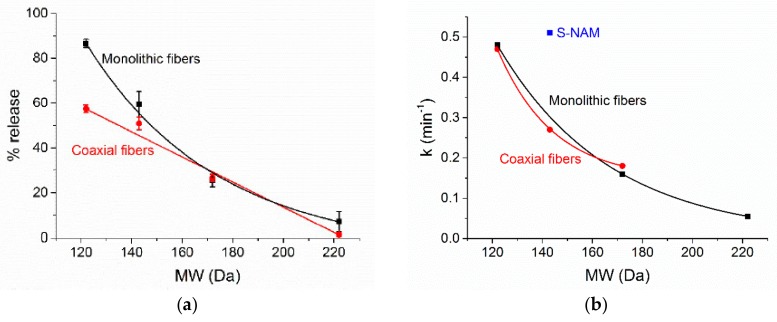
The relationships between AI MW and (**a**) percentage release after 2 h immersion in a pH 1 solution; and (**b**) the Korsmeyer-Peppas rate constant.

It proved possible to develop relationships between the molecular weight and the percentage release attained at pH 1 (Figure 11a). It should be noted that some caution is necessary in extracting these, since they are based on data from only a small number of formulations, but nevertheless some trends are clear. We attempted to fit both simple linear and exponential relationships to the percentage release data. For the monolithic fibers, the best fit to the data is obtained with an exponential relationship taking the form % release = −6.07 + 92.7e^−(MW−122)/51.3^, whereas for the coaxial fibers a more simple linear equation gives the best fit: % release = 126 − 0.561MW. The influence of MW on the release percentage after 2 h at pH 1 is thus dulled by the addition of the blank polymer shell. Considering the Korsmeyer-Peppas rate constants (Figure 11b), an exponential equation of the form k = 0.146 + 0.324e^−(MW−122)/22.3^ gives the best fit to the experimental data for the coaxial systems. In contrast, with the monolithic systems such a relationship can only be constructed for the three carboxylic acids, for which we find k = 0.00372 + 0.476e^−(MW−122)/44.9^. The S-NAM datapoint clearly does not fit on this trendline, indicating that the basicity of the AI has a major influence on the rate constant in the case of the monolithic formulations.

## 4. Discussion

We show in this work that both the molecular weight and the acidity/basicity of an AI are crucial in determining the extent to which release occurs from Eudragit S100 fibers in acidic media. The nanoscale nature of the fiber diameters means that they have very large surface area-to-volume ratios, and thus simply making a fiber from an insoluble polymer does not preclude AI release in conditions where the polymer is insoluble [22,26]. Rather, it is necessary to consider also the ability of the AI to diffuse through the matrix, and the thermodynamic solubility driver for this to happen. It has previously been speculated that lower molecular weight species and those which are more basic will release to a greater extent at acidic pHs [22,25], and in this work we confirm this to be the case.

For coaxial fibers with AI located in the core only, it is demonstrated that the molecular weight of the AI is a good predictor for the percentage and rate of release seen at pH 1, and mathematical relationships can be constructed linking these. For the monolithic fibers, the situation is more complex and although it is possible to elucidate a mathematical relationship between percentage release and AI MW, this does not hold true for the rate of release. For the latter, there is a clear linkage with the MW for the acidic carboxylate AIs considered, but this breaks down for the basic species included in the study. It thus appears that for the coaxial systems, the rate of diffusion through the polymer shell, which will be directly correlated with the MW of the AI, is the dominant factor governing release. For the monolithic analogues, while it is clear that diffusion through the polymer matrix is important, the solubility of the AI in the release milieu is also vital to consider.

## 5. Conclusions

We report new insights into the factors governing the release of active ingredients (AIs) from electrospun Eudragit S100 (ES100) fibers. Monolithic fibers loaded with benzoic acid, 1-naphthoic acid, 1-naphthylamine, and 9-anthracene carboxylic acid were generated, along with analogous systems with an AI-loaded core and an ES100 shell. The fibers were smooth and cylindrical in the main, and comprised amorphous solid dispersions. There was significant release at pH 1 with all of the monolithic fibers except for those containing 9-anthracene carboxylic acid. Both the molecular weight of the AI and its acidity/basicity are important in controlling release from such materials, with lower molecular weight AIs and basic species freed from the fibers more quickly. With the coaxial fibers, since the AI is present only in the core the acidity/basicity appears to be a less important factor, and the molecular weight, and thus the rate of diffusion through the polymer matrix, is found to be the rate limiting step in release. Mathematical equations could be constructed to predict the release obtained in acidic conditions with AIs of differing molecular properties.

## Figures and Tables

**Table 1:** Details of the electrospun formulations prepared in this work.

ID	Active Ingredient (AI)	Theoretical Fiber AI Loading (% *w*/*w*) ^a^	Observed Fiber AI Loading (% *w*/*w*) ^b^	Entrapment Efficiency (%) ^c^	Fiber Diameter (nm)
Monoaxial electrospinning					
S-BA	Benzoic acid	7.69	9.04 ± 0.13	118 ± 2	483 ± 145
S-NA	1-Naphthoic acid	7.69	7.05 ± 1.02	92 ± 13	129 ± 102
S-NAM	1-Naphthylamine	7.69	6.69 ± 0.27	91 ± 4	214 ± 105
S-ACA	9-Anthracene carboxylic acid	7.69	6.54 ± 0.38	85 ± 5	585 ± 158
Coaxial electrospinning					
C-BA	Benzoic acid	2.70	3.42 ± 0.27	127 ± 10	591 ± 189
C-NA	1-Naphthoic acid	2.70	2.38 ± 0.07	88 ± 2	664 ± 171
C-NAM	1-Naphthylamine	2.70	2.20 ± 0.37	81 ± 14	547 ± 124
C-ACA	9-Anthracene carboxylic acid	2.70	2.45 ± 0.16	91 ± 6	621 ± 140

^a^ Calculated based on the relative masses of the polymer and drug in the system. ^b^ Determined experimentally through dissolution of the fibers (mean ± S.D.; *n* = 3). ^c^ Calculated as the percentage of the theoretical loading observed to be incorporated (mean ± S.D.; *n* = 3).

**Table 2:** Values extracted from Kormeyer-Peppas analysis of the AI release data at pH 1.

Formulation	n	k (min^−1^)
S-BA	0.14	0.48
S-NA	0.28	0.16
S-NAM	0.10	0.51
S-ACA	0.36	0.055
C-BA	0.11	0.47
C-NA	0.25	0.18
C-NAM	0.22	0.27
C-ACA	NC	NC

NC = not calculated. The release percentages obtained for C-ACA at pH 1 were all very close to zero, and hence mathematical analysis could not be undertaken.
